# Supplementary material for: Longitudinal Analyses after COVID-19 Recovery or Prolonged Infection Reveal Unique Immunological Signatures after Repeated Vaccinations
Source: Vaccines (Basel). 2022 Oct 28;10(11):1815. doi: 10.3390/vaccines10111815 (PMC9693104; doi:10.3390/vaccines10111815)
Supplement: Supplementary file 1 [file vaccines-10-01815-s001.zip › vaccines-1956122-supplementary.pdf]

Longitudinal Analyses after COVID-19 Recovery or Prolonged Infection Reveal Unique Immunological Signatures after Repeated Vaccinations

Daisuke Hisamatsu [1,*], Akari Ikeda [1], Lisa Ito [1], Yasushi Matsushita [2], Makoto Hiki [3], Hirotake Mori [4], Yoko Tabe [5], Toshio Naito [4] and Chihiro Akazawa [1,*]

[1] Intractable Disease Research Center, Juntendo University Graduate School of Medicine, Tokyo 113-8421, Japan
[2] Department of Internal Medicine and Rheumatology, Faculty of Medicine, Juntendo University School of Medicine, Tokyo 113-8421, Japan
[3] Department of Cardiovascular Biology and Medicine, Juntendo University Faculty of Medicine, Tokyo 113-8421, Japan
[4] Department of General Medicine, Juntendo University Faculty of Medicine, Tokyo 113-8421, Japan
[5] Department of Clinical Laboratory Medicine, Juntendo University Graduate School of Medicine, Tokyo 113-8421, Japan
* Correspondence: d.hisamatsu.ap@juntendo.ac.jp (D.H.); c.akazawa.gt@juntendo.ac.jp (C.A.)

Supplementary Materials

(A)

		WT	WT	WT	Mut
**Severity**	**Healthy**	**Mild**	**Moderate I**	**Moderate II**	**Moderate II**
Number of paticipants	6	10	10	10	8
Age (mean ± SD, years)	46.2 ± 11.5	36.3 ± 17.3	63.7 ± 14.6	70.5 ± 11.3	52.6 ± 7.3
Sex (% male)	50	70	60	60	75
Time from symptom onset to bood collection on admission (mean ± SD, days)	NA	2.9 ± 1.7	5.8 ± 1.2	8.3 ± 4.1	8.0 ± 2.8
Time from admission to discharge (mean ± SD, days)	NA	7.8 ± 5.6	11.1 ± 2.4	14.5 ± 6.3	18.3 ± 5.0
**Clinical factors on admission**					
SpO2 (mean ± SD, %)	NA	97.8 ± 1.4	96.7 ± 1.4	90.9 ± 4.7	94.3 ± 1.7
Fibrinogen (mean ± SD, mg/dL)	NA	317.7 ± 55.8	486.4 ± 102.5	662.0 ± 160.8	533.1 ± 115.0
Ferritin (mean ± SD, ng/mL)	NA	169.0 ± 108.8	629.0 ± 475.2	680 ± 449.2	932.6 ± 300.0
D-dimer (mean ± SD, μg/mL)	NA	1.6 ± 0.4	1.6 ± 0.3	2.5 ± 0.8	1.5 ± 0.2
IL-6 (mean ± SD, pg/mL)	NA	3.6 ± 2.6	22.1 ± 18.3	42.4 ± 32.6	29.3 ± 11.2
Krebs von den Lungen-6 (KL-6) (mean ± SD, U/mL)	NA	238.0 ± 144.5	336.9 ± 469.1	260.0 ± 104.5	256.1 ± 109.7
Serum Amyloid A (SAA) (mean ± SD, μg/mL)	NA	17.6 ± 18.7	386.0 ± 428.5	2029.5 ± 1804.3	346.2 ± 262.7
pulmonary Surfactant Protein-D (SP-D) (mean ± SD, ng/mL)	NA	24.6 ± 8.5	36.1 ± 45.3	57.5 ± 70.4	34.5 ± 41.8
White Bood Cell (WBC) (mean ± SD, x10^3/μl)	NA	5.2 ± 1.8	4.9 ± 1.9	8.0 ± 3.2	4.9 ± 2.3
Hemoglobin (Hb) (mean ± SD, g/dL)	NA	15.1 ± 1.8	13.7 ± 1.5	13.3 ± 0.5	15.0 ± 0.8
Neutrophil (NE) (mean ± SD, %)	NA	59.9 ± 11.5	68.7 ± 9.8	81.5 ± 11.3	71.5 ± 9.7
Lymphocyte (Lym) (mean ± SD, %)	NA	28.2 ± 9.0	22.7 ± 8.8	11.8 ± 8.8	20.1 ± 7.7
Monocyte (Mo) (mean ± SD, %)	NA	8.7 ± 3.1	7.7 ± 2.4	5.3 ± 3.2	7.8 ± 2.5
Eosinophil (Eos) (mean ± SD, %)	NA	2.8 ± 2.7	0.7 ± 0.8	0.2 ± 0.3	0.1 ± 0.2
C-Reactive Protein (CRP) (mean ± SD, mg/dL)	NA	0.3 ± 0.1	4.4 ± 5.3	11.6 ± 7.2	5.0 ± 3.5

(B)

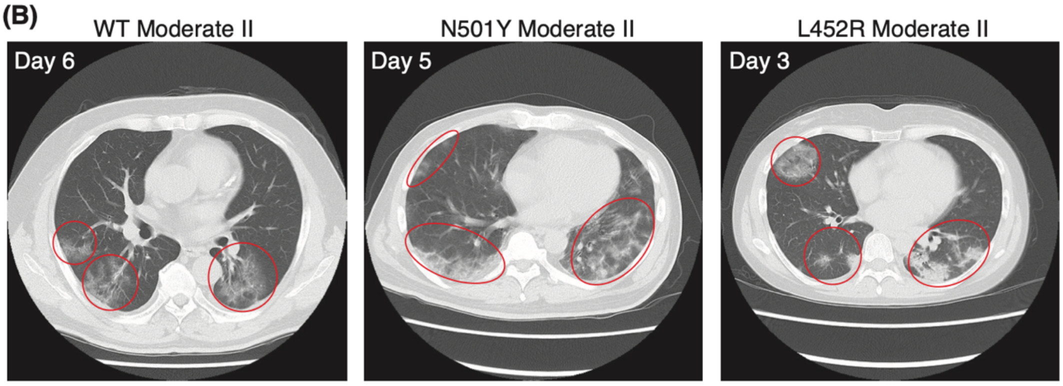

Supplementary Figure S1. Study cohort. (A) Clinical information of all participants. WT indicates the original SARS-CoV-2 strain, and Mut indicates the VOCs strain, including Alpha and Delta strains. (B) Representative images of COVID-19 pneumonia in moderate II patients. Red circles show lesions.

Group	P#	G-CSF	GM-CSF	IFN-α	IL-1α	IL-2	IL-4	IL-5	IL-6	IL-8	IL-10	IL-12p40	IL-13	IL-17A	MCP-1	MIG	MIP-1α	RANTES	Eotaxin	TNF	FasL	I-TAC	IP-10
WT Mild	1-A								6.58	11.5		24.09		3.22	116.49	106.04	8.3	16227	55.14		20.12		239.50
	3-A	0.36	2.47	4.81	0.61			0.41	12	21.72	2.41	130.37		2.6	64.35	69.63	2.14	5405.2	51	3.8	49.20	78.93	290.31
	3-D	0.36	1.3					2.45	31.03	21.72	4.39	135.25			68.93	50.69	8.3	2605.3	48.15	11.91	43.86	47.36	290.71
	4-A		3.94		3.1		9.99		5.48		5.24	150.06		10.25	426.42	32.15	39.96	8658.7	26.81		34.48		89.46
	4-D	1.61	0.71		1.22			1.37				125.51		15.3	8.81	27.85	43.56	14644	31.46	572.68	32.16		59.77
	5-A	1.26		5.54					5.85			52.28		1.21	156.53	73.45	2.9	6442.1	48.15	1.67	42.09	35.93	372.62
	5-D		0.13		1.84		7.21		3.17			101.64		4.72	78.93	52.6	1.88	4638	31.46	1.67	40.91		145.84
	6-A	2.63		1.11	0.31			2.34	6.58	2.97		106.35			189.79	107.77	0.87	24249	28.04		31.01		491.45
	6-D	0.91		1.87	5.06		8.61	0.41	5.85	17.45	4.39	145.1		1.21	196.01	98.96	4.68	16182	28.66	6.81	35.64		319.00
	7-A	0.54		32.67	1.22		4.2	1.12	17.76	21.36	4.82	115.87		5.6	856.25	95.78	95.71	30370	51.64	92.25	26.41	538.32	3760.38
	7-D				0.91			0.85	2.36		3.47			4.13	185.64	50.5	16.91	23167	25.88	17.88	18.41		65.59
	8-A	1.26	0.13	5.91					13.44	25.7	6.05	0.05		1.94	276.96	126.95	10.68	9264	53.55	5.39	23.55	8.58	487.62
	9-A	0.36		17.49	0.61			1.12	6.95	4.1	5.24	175.17		1.94	13.76	201.01	0.62	941.37	18.54	143.17	52.79	23.52	743.90
	9-D	0.54	0.71		1.22				3.96			20.35		0.19	115.24	90.3	2.9	7370.7	24.95		40.91		135.65
	10-A			0.33			4.2	0.41	1.02	4.83		48.07		2.6	68.09	19.78	3.91	12744	20.97		21.83		267.83
	10-D		1.88		0.31		7.21		4.34	0.9		48.07		0.19	59.36	33.23	4.17	14969	24.03	1.67	20.69		82.87
WT Moderate I	11-A	2.12		38.91	0.31			0.23	28.79	40.26	9.42	200.75		4.43	276.55	213.88	5.45	7239.2	17.03		22.03	144.42	2355.23
	11-D		3.64		0.91			0.23	8.04	11.15				3.22	118.58	209.82	4.17	16042	7.6		15.44		355.23
	12-A	3.46		22.97	4.39			1.74	36.31	6.97		60.86	12.93		168.18	169.89	3.4	12639	46.56	11.91	30.21	56.33	1365.64
	12-D			7				0.56	5.1	0.42		6.34			179.82	80.57	2.64	21579	57.37	11.3	10.84		99.24
	13-A	1.26	3.35	9.94				0.98	21.4	14.99		101.64		4.43	152.78	150.73	4.93	9111	40.24	6.81	16.96	13.77	760.16
	14-A	3.13		76.66	0.91			0.71	49.78	46.03	13.31	140.16		3.83	222.93	262.3	5.19	13558	33.96		7.11	189.37	2129.52
	14-D	0.17					2.46	2.22	6.95	36.46	1.76	9.68		3.83	150.28	116.51	16.36	8216.8	64.08	17.88	8.74		190.60
	15-A	5.57			1.22		4.2	0.56	22.13	39.49		120.68			114.41	142.86	10.94	10488	46.24	11.91	21.52		513.54
	16-A	12.64		3.35					10.92	5.19	6.83	3.12		1.94	74.76	166.54	0.87	4708.9	19.76	6.11	20.00	34.91	977.28
	17-A	1.43	22.59	10.31	0.31	101.84		0.56	9.84	4.1		16.69		2.6	227.48	69.03	2.14	8259.3	76.88	2.87	38.00	3.22	1283.75
	17-D	1.95	19.37			63.14			1.93	6.61					138.19	23.97	10.15	15019	77.84	3.8	20.00		115.66
	18-A	1.43	1.88		0.31		5.75		34.42	17.1	2.41	78.59		4.72	56.87	228.51	8.83	15832	36.47	4.62	20.00		1296.57
	18-D			1.5					3.57	0.9		74.09		3.22	43.63	93.45	3.15	7839	44.98	7.49	20.50		127.16
	19-A	1.78	0.71	420.14			6.73		69.16	61.06	24.32	657.05			200.16	1208.2	10.94	22412	54.82	11.3	35.91	359.95	5611.97
	19-D	2.96			1.84		4.2	3.82	14.15	34.57	2.41	200.75		5.02	124.43	1680.1	13.63	24842	65.67	2.87	22.03	24.31	491.99
	20-A	1.78	15.87	23.36		102.3	0.99		26.19	9.06	6.44	150.06		3.53	141.95	167.26	2.9	14686	50.37	3.8	28.15	78.04	1908.26
	20-D	1.95	20.65			92.02			9.48	13.59	10.5			3.53	94.78	186.14	5.45	12992	55.46	6.11	22.03	24.31	1327.30
WT Moderate II	21-A	0.17			4.07		3.65	1.62	34.42	20.65	0.87	130.37	2.04	2.91	70.18	44.83	7	4734.5	54.5	11.3	44.87		197.12
	23-A	22.45	35.14			54.86		1.37	502.55	30.84	50.23	43.91		0.19	433.31	214.9	12.01	9629.5	44.34	5.39	16.96	302.93	7215.05 †
	23-D	115.7	3.94					0.41	544.61	800.92	20.2	0.05	0.21	28.48	599.83	1122.9	42.05	8022.8	63.76	8.79	24.06	111.03	458.68
	24-A	4.28		5.18		17.72	4.2	1.74	5.85	33.07	5.24	87.7		0.19	51.89	216.68	6.22	7076.2	24.95	8.15	21.01	200.67	2995.72 †
	24-D	2.29	5.42		3.42			2.22	30.65	54.67					397.17	209.06	9.62	13211	47.19	3.8	10.32	34.91	925.89
	25-A	4.28	1.01	0.73			5.25	0.41	42.81	12.89	2.41	39.81			417.08	1170.3	12.01	9200.5	48.15	8.15	23.55	144.42	5420.84
	25-D	0.54	1.01		1.22			0.56	8.04	9.06		69.64		2.91	299.56	212.1	9.09	13922	67.91		20.00		198.89
	26-A	1.26							11.28	17.8	4.39				19.87	853.96	6.48	20079	20.36	5.39	13.41	34.91	1214.38
	26-D	4.6			1.53			0.41	23.97	27.16	12.96			3.83	181.9	272.58	6.22	4408.6	42.13	3.8	17.97		496.41
	27-A	3.3	1.3					0.85	5.1	15.34		27.91			27.2	120.04	4.42	16902	23.73		8.20		375.07
	27-D				0.91				5.85	3.35		96.96		3.22	82.68	59.37	3.91	17542	46.88		16.96		154.85
	28-A	1.43	1.88				5.25	0.41	23.97	74.91	1.76	60.86		5.02	152.78	147.48	11.48	8723.5	50.05	4.62	9.27		248.21
	28-D	2.63	1.01				2.46	1.86	16.68	31.58	2.41	216.31			170.26	368.97	2.14	13841	74.96	6.81	6.55		225.40
	29-A	1.08		0.33	1.53			2.1	39.74	19.93	10.85	20.35			92.7	177.36	3.66	6612	26.81		11.87	189.37	2066.70
	29-D	1.43	1.01		1.22			3.48	17.4	15.69		115.87	9.95	3.53	197.26	281.32	8.56	15888	48.15		17.97	189.37	326.73

Expression level

high (pg/mL) low N.D

Supplementary Figure S2. Comprehensive serum cytokine analysis in COVID-19 patients infected with WT. Table shows the concentration of each serum cytokine. P# indicates patient identification numbers (*n* = 9 patients/WT-infected mild patient group; 10 patients/WT-infected moderate II patient group; 8 patients/WT-infected moderate II patient group). A indicates admission and D indicates discharge. Crosses show deceased patients.

Mut Moderate II

P#	G-CSF	GM-CSF	IFN-α	IFN-γ	IL-1α	IL-2	IL-4	IL-5	IL-6	IL-8	IL-10	IL-12p40	IL-13	IL-17A	MCP-1	MIG	MIP-1α	RANTES	Eotaxin	TNF	FasL	I-TAC	IP-10
33-A	4.34	1.66							7.03	28.51	6.34	44.70	1.34		62.54	859.44	0.34	9665.29	26.24		16.50	149.21	2354.14
33-D	0.01	1.13								31.07	3.89	14.84			114.20	151.86	12.78	8147.68	70.00	1.96	11.65	61.65	555.66
34-A	0.26	0.01							54.23	295.06	0.01				144.89	433.08	51.20	25957.46	51.80	19.11	16.50	71.66	582.22
34-D						7.50			3.73		2.24	37.73		10.95	144.89	191.13		482.13	63.05		15.13	41.65	58.78
35-A	11.92	0.58	7.10							15.24	8.00	30.60	1.34	2.24	100.52	230.90		13395.63	74.00		11.65	76.68	577.22
35-D										10.07	0.95		3.17	1.21	134.48	211.77	3.86	5487.11	150.82		15.13	41.65	71.57
36-D		0.58							9.28	46.79	3.89	156.32		3.75	177.90	371.12	7.44	25580.16	71.72		24.58	81.72	265.54
37-A	1.52	4.26	39.59	3.08					22.85	15.87	10.52	120.67	3.17	1.73	414.34	470.71	1.51	14837.48	53.00		30.59	96.97	1355.98
37-D	1.02			1.26						6.60	3.89	92.75	0.88	0.68	222.43	218.09	4.26	23567.54	85.24		29.25	51.67	243.82
38-A	1.52		40.36						35.98	45.46	5.52			4.76	383.79	144.34	2.69	14740.33	93.51		17.18	61.65	893.57
38-D				2.52					6.25	27.24	3.07		0.42		453.16	138.78	2.30	20629.67	134.13		8.00	46.67	117.12
39-A	4.34	2.19	64.16	8.14	0.03	9.55			48.05	17.76	13.96	14.84	2.25	1.21	147.11	279.80	1.12	8051.89	40.81		17.18	81.72	1678.94
39-D			3.90		3.50					6.60	8.00		0.88	3.75	218.16	94.98		10496.13	80.22		24.58	36.59	60.92
40-A	3.05		88.25	3.08					74.84	20.28	15.27	156.32	3.17	2.75	158.91	1744.91	1.12	7615.13	72.86		34.60	122.81	2935.03
40-D	1.52				5.87	7.50		0.27	12.17	14.60	4.70	51.59	0.88	2.24	156.70	822.49		8932.56	63.05		24.58	46.67	117.54

Expression level

high (pg/mL) low N.D

Supplementary Figure S3. Comprehensive serum cytokine analysis in COVID-19 patients infected with VOCs. Table shows the concentration of each serum cytokine. P# indicates patient identification numbers (*n* = 8 patients/VOC-infected moderate II patient group). A indicates admission and D indicates discharge.

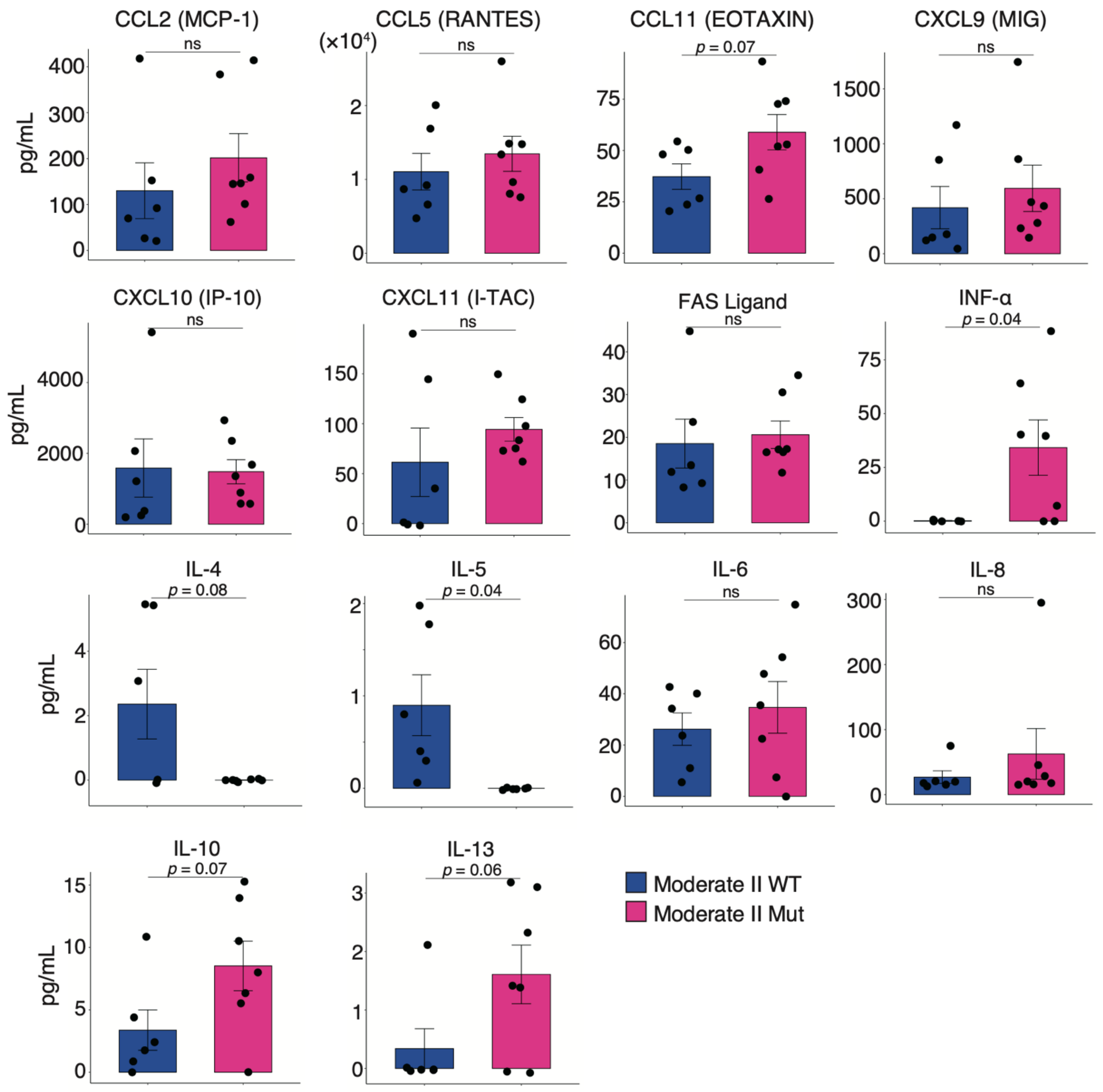

Supplementary Figure S4. Comparison of serum cytokine levels in moderate II patients infected with the WT strain or VOCs on admission. Graphs show the concentration of each cytokine (n = 6 patients/WT-infected moderate II patient group; 7 patients/VOC-infected moderate II patient group). Statistical significance was determined using Welch's t-test. ns, not significant.

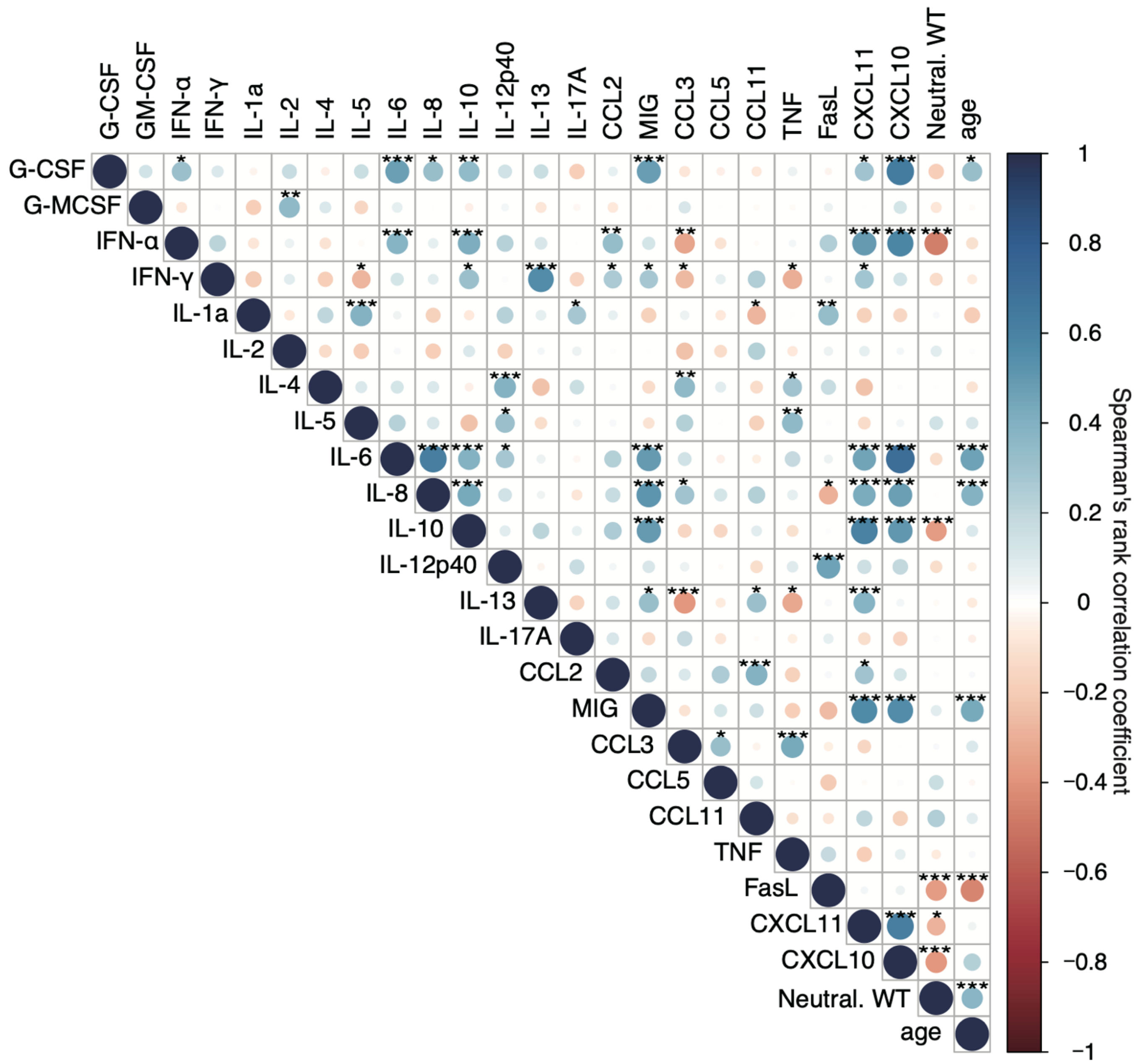

Supplementary Figure S5. Correlation analysis among serum cytokine level, neutralizing antibody titer, and age in COVID-19 patients during hospitalization period. Correlation matrices were created using the Spearman's correlation coefficient (n = 33 patients). Neutral. WT, neutralizing potency for WT strain; *p < 0.05; **p < 0.01; ***p < 0.005.

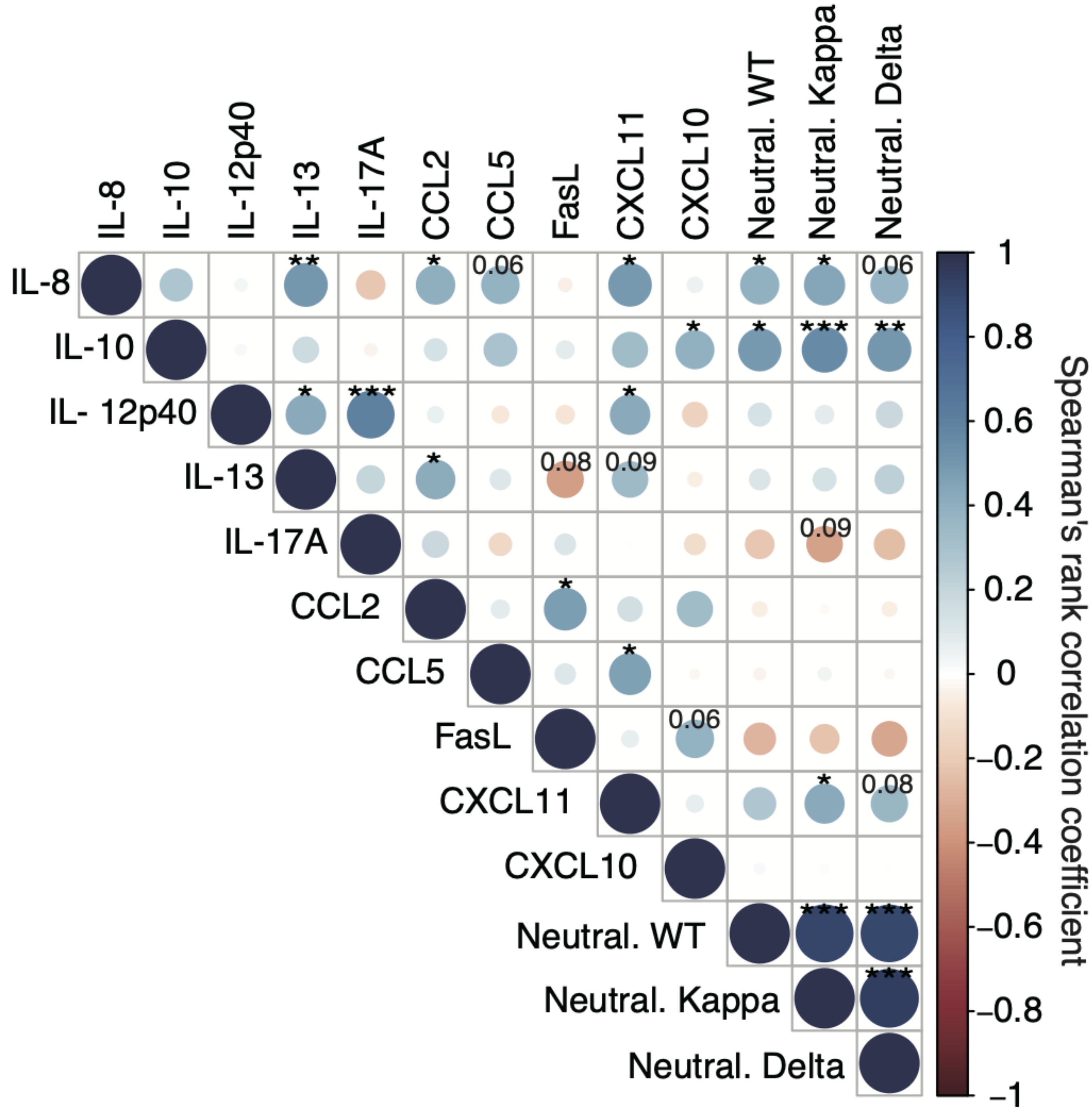

Supplementary Figure S6. Correlation analysis of serum cytokine level and neutralizing antibody titer in all participants, including naïve and recovered individuals. Correlation matrices were created using the Spearman's correlation coefficient (n = 10 participants). Neutral. WT, neutralizing potency for WT strain; Neutral. Kappa, neutralizing potency for Kappa strain; Neutral. Delta, neutralizing potency for Delta stain; *p < 0.05; **p < 0.01; ***p < 0.005.

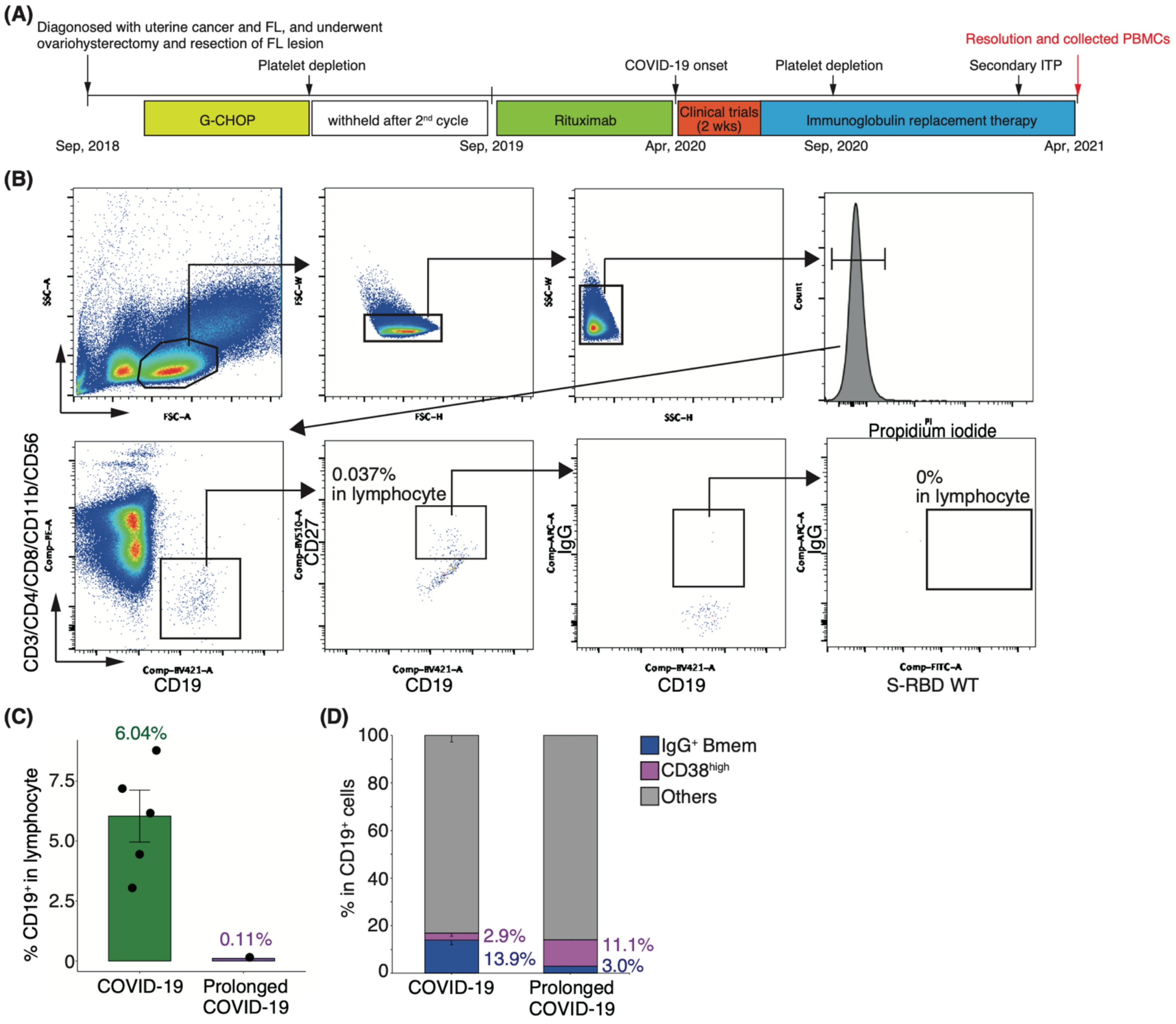

Supplementary Figure S7. Prolonged SARS-CoV-2 infection in a patient with follicular lymphoma undergoing B-cell depletion therapy. (A) Graph showing the clinical course and treatment strategy. FL, follicular lymphoma; ITP, immune thrombocytopenia; G-CHOP, obinutuzumab plus cyclophosphamide, doxorubicin, vincristine, and prednisone therapy. (B) Representative FACS plots of the cell population are shown in panels. The number indicates the positive rate. (C) The graph shows the proportion of CD19$^+$ cells in each group during recovery (n = 5 patients/COVID-19 group). (D) Graph showing the frequencies of IgG$^+$ Bmems

and $CD38^{high}$ cell populations during recovery (*n* = 5 patients/COVID-19 group). Each number indicates the mean value.

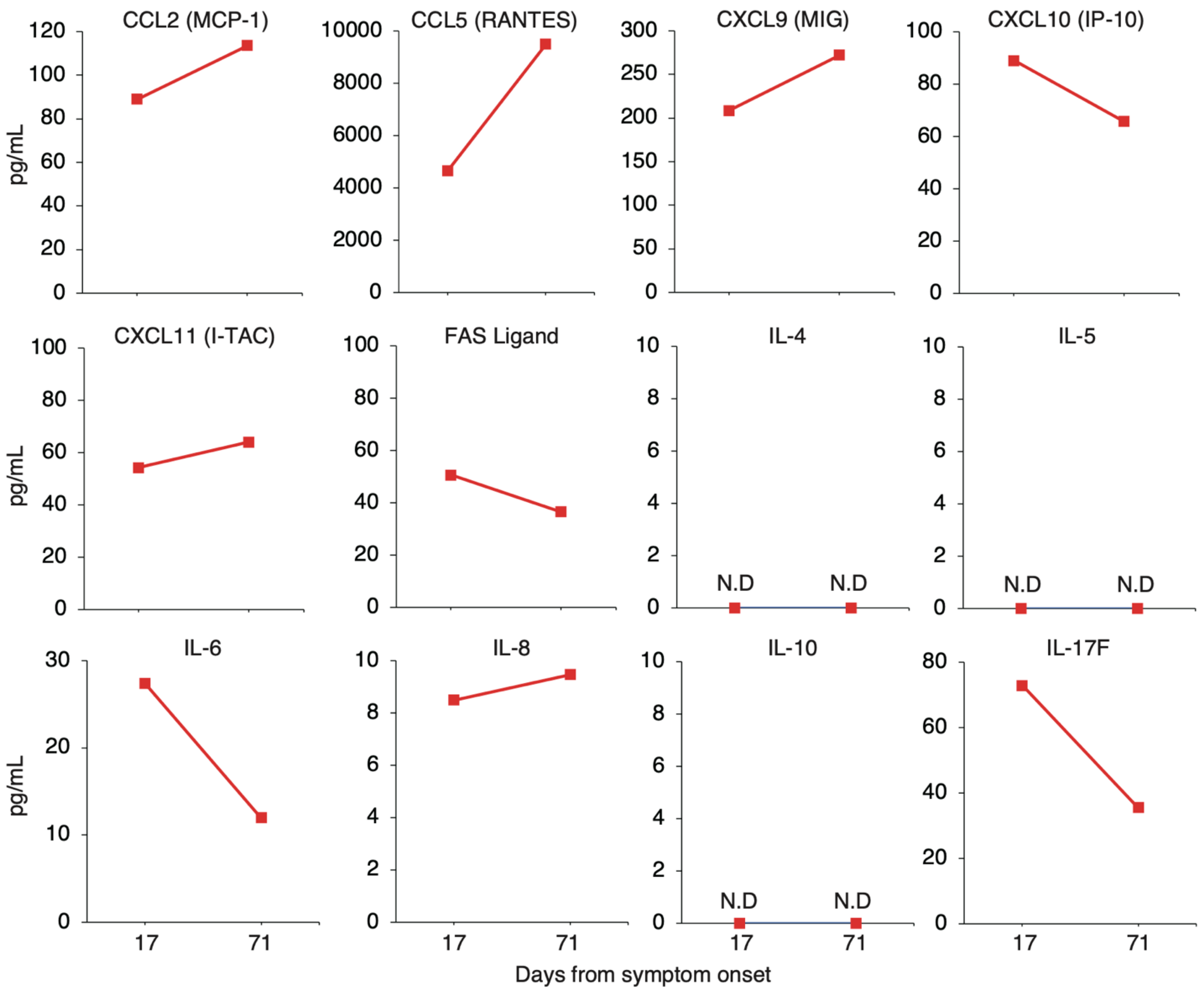

Supplementary Figure S8. Serum cytokine level change in a patient with prolonged COVID-19. Graphs show representative cytokine concentrations. N.D, not detected.

Supplementary Table S1. Primer sets of single-cell RT-PCR and immunoglobulin gene sequencing.

Forward Primer

Target	Sequence
IGH_variable region (v) 1	5-ACAGGTGCCCACTCCCAGGTGCAG-3
IGH_v3	5-AAGGTGTCCAGTGTGARGTGCAG-3
IGH_v4/6	5-CCCAGATGGGTCCTGTCCCAGGTGCAG-3
IGH_v5	5-CAAGGAGTCTGTTCCGAGGTGCAG-3
IGL(κ)_v1/2	5-ATGAGGSTCCCYGCTCAGCTGCTGG-3
IGL(κ)_v3	5-CTCTTCCTCCTGCTACTCTGGCTCCCAG-3
IGL(κ)_v4	5-ATTTCTCTGTTGCTCTGGATCTCTG-3
IGL(λ)_v1	5-GGTCCTGGGCCCAGTCTGTGCTG-3
IGL(λ)_v2	5-GGTCCTGGGCCCAGTCTGCCCTG-3
IGL(λ)_v3	5-GCTCTGTGACCTCCTATGAGCTG-3
IGL(λ)_v4/5	5-GGTCTCTCTCSCAGCYTGTGCTG-3
IGL(λ)_v6	5-GTTCTTGGGCCAATTTTATGCTG-3
IGL(λ)_v7	5-GGTCCAATTCYCAGGCTGTGGTG-3
IGL(λ)_v8	5-GAGTGGATTCTCAGACTGTGGTG-3

Reverse Primer

Target	Sequence
IGH_constant region (c) 1	5-CGCCTGAGTTCCACGACACC-3
IGH_c for nested PCR	5-TCGGGGAAGTAGTCCTTGAC-3
IGL(κ)_c1	5-GAGGCAGTTCCAGATTTCAA-3
IGL(κ)_c for nested PCR	5-GGGAAGATGAAGACAGATGGT-3
IGL(λ)_c1	5-GCTTGAAGCTCCTCAGAGG-3
IGL(λ)_c for nested PCR	5-GGGCGGGAACAGAGTGACC-3
